# Recognizing Growth Concerns in 1- to 5-Year-Olds: A Practical Algorithm for Screening and Early Intervention in Primary Care

**DOI:** 10.3390/children13050654

**Published:** 2026-05-07

**Authors:** James Best, Peter S. W. Davies, Laura Hunt, Andrew Leech, Helen McCarthy, Tim Warnock

**Affiliations:** 1 Junction Street Family Practice, Nowra 2541, Australia; 2 Child Health Research Centre, University of Queensland, Brisbane 4101, Australia; ps.davies@uq.edu.au; 3 Family Circle Medical Practice, Newcastle 2303, Australia; lhun3844@outlook.com; 4 The Garden Family Medical Clinic, Perth 6150, Australia; andrew@thegardenmedical.com; 5 School of Medicine, University of Notre Dame, Fremantle 6160, Australia; 6 Institute of Health and Sport, Victoria University, Melbourne 8001, Australia; helen.mccarthy1@vu.edu.au; 7 Cairns Paediatrics, Cairns 4870, Australia; thwarnock@gmail.com

**Keywords:** childhood growth, growth screening, growth velocity, overweight, obesity, wasting, stunting, primary care, general practice

## Abstract

**Background:** Poor or rapid growth velocity in early childhood can have negative long-term health consequences. Early identification of children at risk of growth problems facilitates timely intervention to change growth trajectories and health parameters, but barriers exist to routine screening in primary care. **Methods:** Experts from general practice, primary care nursing, pediatrics, dietetics, and academic research convened to develop a practical algorithm to support routine growth screening and intervention in children aged 1 to 5 years in primary care. **Results:** Using a single initial measurement of weight and height in conjunction with risk factor assessment, the algorithm presented here identifies children at risk of poor or rapid growth velocity and provides guidance for intervention and follow-up frameworks. **Conclusions:** This tool helps to define children in the community setting who are at risk of poor or rapid growth velocity and supports primary care providers in promoting healthy growth trajectories through timely intervention and continued routine growth monitoring.

## 1. Introduction

The first 1000 days of life, from conception to 2 years of age, are critical for children’s physical growth and brain development [1,2]. However, the next 1000 days, from 2 to 5 years of age, are also a crucial period to improve growth and development trajectories in children who may be at risk of growth problems [2].

Abnormal growth velocity in early childhood can have significant impacts on physical, mental, and social health [3,4]. Poor growth velocity can lead to wasting and/or stunting, which is associated with increased risk for infections, poor physical and cognitive development, and ultimately non-communicable diseases [5,6]. Rapid growth velocity, notably weight velocity, can drive overweight and obesity [7], which increases risk for continued obesity and associated cardiometabolic disorders into adolescence and adulthood [8,9].

Among children aged 1 to 5 years in Australia, recent nationally representative data on growth velocity are limited, with most studies focusing on prevalence of wasting/stunting or overweight/obesity in children in inpatient settings or specific community-based populations (e.g., refugees, remote Indigenous populations) [10,11,12,13]. Data are lacking to demonstrate the scale of growth velocity problems in the broader Australian community setting and the proportion of children in this age group who may be at risk of poor or rapid growth velocity.

Identifying early deviations from normal growth trajectories could offer key opportunities to intervene before children reach a diagnosis of wasting/stunting or overweight/obesity. However, barriers to identifying early signs of risk persist, including challenges related to current growth screening and monitoring practices used in Australia [14,15,16]. Australian guidelines recommend growth monitoring for children up to 6 years of age at ‘well child’ or immunization visits [17], but structured programs beyond 18 months of age and centralized systems for tracking measurements are lacking. Even within structured programs that continue beyond infancy, evidence in Australia and globally suggests that the proportion of children participating in routine health screening decreases with increasing age [18,19]. Disjointed health services, and significant time and resource restraints increasingly faced by general practitioners (GPs), contribute to inconsistency in timing and frequency of child growth monitoring in primary care [14,15,16]. As a result, routine growth monitoring is often deprioritized or overlooked during GP consultations.

Identifying changes in growth velocity relies on tracking serial weight and height assessments over time; however, cost and time pressures may limit families’ ability to commit to return visits for follow-up measurements [15]. Patients may not see the same provider each visit, introducing potential inconsistency in how growth data are obtained. Importantly, infrequent visits or inadequate follow-up could delay early identification and timely intervention for children at risk of growth problems, with a negative impact on ongoing health.

While appropriate use of growth charts can support effective ongoing pediatric growth monitoring [17], there are currently no published and validated tools to support routine growth screening in primary care in Australia. To support routine growth screening in children aged 1 to 5 years and encourage early identification and intervention of those at risk for poor or rapid growth velocity, we developed an algorithm designed to provide a structured framework for implementation in primary care settings.

## 2. Development of the Pediatric Growth Screening Algorithm

The proposed pediatric growth screening algorithm was developed using expert consensus underpinned by a narrative literature review.

### 2.1. Expert Selection Process

To support development of the proposed algorithm, an advisory board was convened in April 2025. Their objective was to align on strategies to address barriers to routine growth screening in children aged 1 to 5 years in primary care settings and identify those at risk of poor or rapid growth velocity, for whatever underlying reasons.

Advisory board members were selected based on predefined criteria to provide relevant expertise that aligned with the study objectives and to provide multidisciplinary representation across key primary care disciplines involved in routine growth monitoring. Selection criteria included clinical or academic expertise in pediatric growth, nutrition, or primary care; current clinical practice or research experience with children aged 1 to 5 years; and experience within the Australian healthcare context. Potential advisors were identified through professional networks including the Child and Young Person’s Health Specific Interest Group of the Royal Australian College of General Practitioners, the Australian Primary Healthcare Nurses Association, and the Australian Paediatric Society, as well as through peer-reviewed publications, clinical leadership roles, and involvement in child health guideline development or education.

The final advisory board brought together experts from general practice, primary care nursing, pediatrics, dietetics, and academic research across four Australian states. The multidisciplinary and geographic diversity of advisors was designed to reflect real-world primary care practice and support development of a pragmatic, implementable algorithm.

### 2.2. Literature Review Parameters

A narrative literature review was conducted to investigate routine growth monitoring practices in children and risk factors for and rates of overweight/obesity and underweight in children. To identify relevant literature, a search was performed in PubMed and Google Scholar using key search terms including, but not limited to, ‘growth’ AND ‘monitoring’ AND ‘children’, and ‘growth’ AND ‘overweight’ OR ‘obesity’ OR ‘underweight’. Results were limited to English-language, peer-reviewed journals with articles published in the past 10 years, and those with Australian context were prioritized.

### 2.3. Consensus Methodology

Prior to the first advisory board meeting, advisors independently completed a pre-meeting questionnaire to provide insights into current growth screening practices in Australia, key measurements used, criteria for identifying children at risk of altered growth velocity, associated risk factors, and perceived barriers and potential solutions to encourage routine growth screening and early intervention. All responses were reviewed and discussed during the facilitated advisory board meeting in April 2025.

Following these discussions, the advisors identified that a practical clinical tool could help address some of the identified limitations of current growth screening practices. They developed an algorithm based on supporting literature and their own clinical experience and expert opinion, which was reviewed and refined in a subsequent advisory board meeting held in June 2025. Subsequent rounds of review were conducted to reach consensus on the final pediatric growth screening algorithm presented here.

## 3. Proposed Pediatric Growth Screening Algorithm

**Figure 1:**
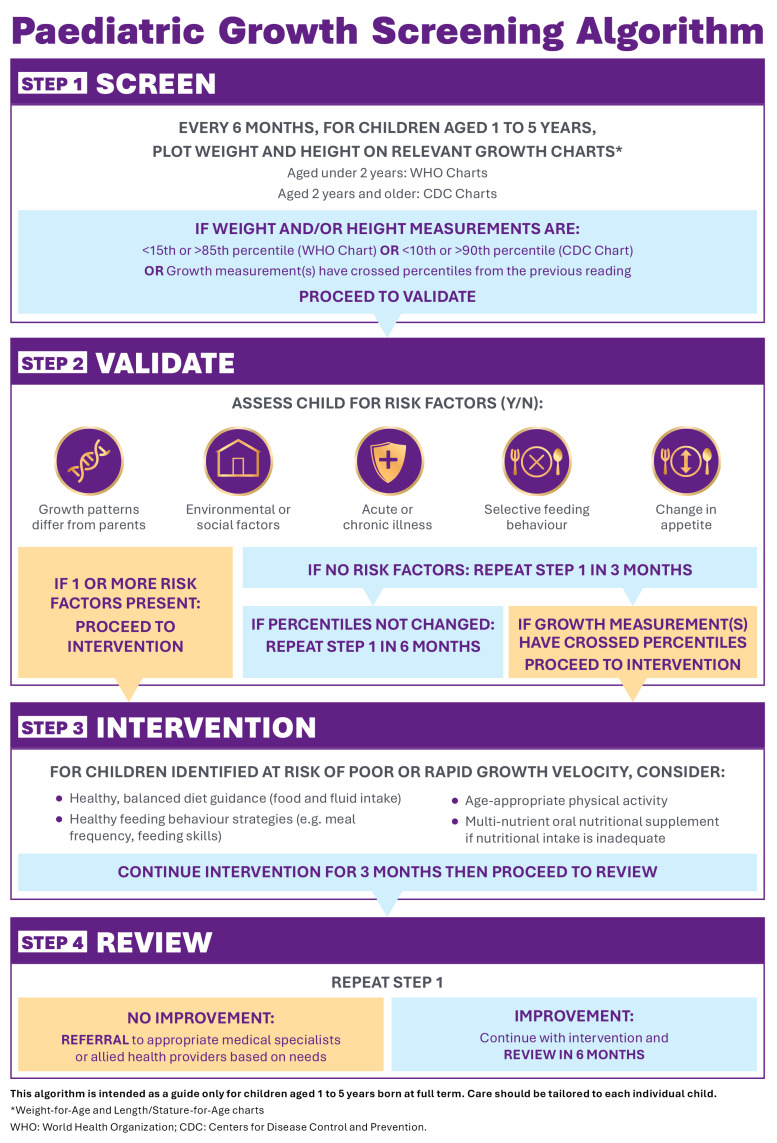
The pediatric growth screening algorithm for children aged 1 to 5 years in the primary care setting.

The proposed algorithm provides guidance across four steps: screen, validate, intervention, and review (Figure 1). It uses an initial single measurement of weight and height, with values outside a specified range prompting assessment of specific risk factors to determine whether intervention is required. The proposed recommendations aim to ensure that children return for timely follow-up and provide a framework to obtain serial measurements to track growth velocity over time and were informed via pre-meeting and post-meeting questionnaires.

### 3.1. Step 1: Screen

The algorithm recommends measuring weight and height at least every 6 months in children aged 1 to 5 years old, which helps increase touchpoints in primary care between immunizations at 18 months and 4 years of age [17]. When screening, providers (GPs, practice nurses, or child health nurses) should use calibrated, age-appropriate equipment and best practice techniques to ensure accurate and consistent measurements [20]. Measurements should be plotted on the relevant growth chart identified in the algorithm (Figure 1) for the child’s age and sex [17,21,22]. Children who fall into the categories described in the algorithm (Figure 1) should proceed to assessment in Step 2. Children who do not fall into these categories should return for routine review in 6 months.

The thresholds selected to proceed to Step 2 represent approximately one standard deviation (SD) from the mean (±1.036 SD when using the 15th and 85th centiles; ±1.282 SD when using the 10th and 90th centiles) [21,22]. While values outside these centiles in isolation may not indicate growth problems, they may signal a child is at risk if combined with other factors. Crossing centiles upward or downward from a previous measurement can flag potential growth problems, but these children should also be reviewed for additional risk factors due to the nonlinear nature of growth trajectories in early childhood [20,23]. This algorithm uses growth charts designed for term infants and children, so it should not be applied to children born prematurely (before 37 weeks’ gestation), who may have impacted growth trajectories into early childhood [24], or other children who should not be tracked on standard growth charts.

### 3.2. Step 2: Validate

Assessing for certain contributing risk factors can help validate whether a child identified using the centiles described in the algorithm is at risk of poor or rapid growth velocity or maintaining a normal growth trajectory. Growth patterns that differ from parents’, environmental or social factors, acute or chronic illness, selective feeding behaviors, and appetite changes were identified as potential risk factors for growth problems in children aged 1 to 5 years [20,25,26,27,28]. Children with one or more risk factors present should proceed directly to intervention. Children with none of these risk factors should have their weight and height reassessed in 3 months and only proceed to intervention if their measurements have crossed centiles in the intervening time.

### 3.3. Step 3: Intervention

This algorithm defines children at risk of poor or rapid growth velocity as those outside the 15th to 85th (<2 years) or 10th to 90th centiles (2–5 years) or crossing centiles since a previous assessment, with at least one additional risk factor present. Identifying ‘at-risk’ children provides an opportunity for early intervention with evidence-based strategies to improve diet and nutritional intake, healthy feeding behaviors, and physical activity [29,30]. Family education and nutrition counseling, particularly around healthy balanced food intake and activity levels, were considered important early interventions in at-risk children. Providers should supply families with relevant handouts or reputable online resources and consider referral to a suitable allied health provider. Providers may also wish to consider multi-nutrient child oral nutritional supplements for children unable to meet nutritional requirements with food alone, including those at risk of poor growth velocity, to supplement overall energy intake [30]. These supplements may also benefit children at risk of rapid growth velocity, who can experience nutritional deficiencies due to diets high in calorie-dense but nutrient-poor foods [31].

### 3.4. Step 4: Review

Three months was considered an appropriate follow-up interval, allowing sufficient time to assess the effect of the intervention on growth metrics without waiting too long between visits. For children who show improved growth metrics, continued interventions as appropriate with routine 6-monthly reviews are recommended, ideally with the same primary care provider to ensure continuity and consistency of measurements. Recall systems within practice software could be used to support this process. For children who do not show improved growth metrics, providers should consider referral to a pediatrician or other appropriate allied health services.

## 4. Discussion

This algorithm is designed as a practical tool for use in primary care settings by GPs and nurses, providing a conceptual framework to develop routine growth screening practices and obtain serial measurements to support long-term growth trajectory monitoring. By defining at-risk children, it offers a simple framework to identify those who require intervention to modify growth trajectory and improve health outcomes.

At present, the algorithm has not been formally validated in clinical practice; however, we believe the underlying scientific and clinical principles are sound. As this algorithm becomes utilized in primary care, ongoing assessment of its feasibility and validity in practice can be undertaken. These assessments will help provide understanding of barriers and enablers to its practical application in different primary care settings across Australia and in which situations the algorithm may be particularly beneficial (e.g., patients with limited access to GPs and practices with primary health care nurses). Future studies are planned to assess the sensitivity, specificity, and predictive value of the defined centile thresholds and contributing risk factors for identifying at-risk children.

To support implementation of the algorithm within primary care practices, targeted education and training for GPs and nurses in primary care settings are recommended [15,32]. Ideally, a digital version of the algorithm would support accessibility for providers and automation for timely identification of growth deviations and follow-up reminders [33]. The costs of implementing the algorithm in primary care practices are currently unknown; however, the basic equipment for taking and plotting weight and height measurements is standard in community-based healthcare settings. Cost-effectiveness analyses could be considered in future feasibility studies.

In striving for simplicity as a screening tool, the proposed algorithm is not designed as a diagnostic tool. However, identifying children at risk of growth concerns via this algorithm provides the opportunity to initiate appropriate follow-up examinations and/or testing to elucidate any underlying pathologies, if present. Furthermore, by providing a common screening and intervention tool for use across Australian primary care settings, particularly if digitized, opportunities arise to capture growth data to fill knowledge gaps around prevalence and risk factors contributing to growth concerns in this community-based population of young children [33].

## 5. Conclusions

Intervention at the earliest signs of poor or rapid growth velocity in early childhood is only possible when children at risk can be identified. The proposed algorithm helps define which children aged 1 to 5 years in the community setting are at risk of poor or rapid growth velocity. Implementing this algorithm in primary care can help address gaps in current growth screening practices and educate providers to take a proactive role in child growth monitoring. It also supports increased opportunities to track growth velocity with serial measurements and to intervene at early signs of risk to help change growth trajectories and long-term health outcomes.

## Data Availability

No new data were created or analyzed in this study. Data sharing is not applicable to this article.

## Abbreviations

The following abbreviations are used in this manuscript:

GP	General Practitioner
SD	Standard deviation
